# Perception of temporal synchrony not a prerequisite for multisensory integration

**DOI:** 10.1038/s41598-024-55572-x

**Published:** 2024-02-29

**Authors:** Robert M. Jertberg, Sander Begeer, Hilde M. Geurts, Bhismadev Chakrabarti, Erik Van der Burg

**Affiliations:** 1 Department of Clinical and Developmental Psychology, The Netherlands and Amsterdam Public Health Research Institute, Vrije Universiteit Amsterdam, Amsterdam, The Netherlands; 2 Brain and Cognition, Department of Psychology, Dutch Autism and ADHD Research Center (d’Arc), Universiteit van Amsterdam, Amsterdam, The Netherlands; 3 Centre for Autism, School of Psychology and Clinical Language Sciences, University of Reading, Reading, UK; 4 India Autism Center, Kolkata, India; 5 Department of Psychology, Ashoka University, Sonipat, India

**Keywords:** Human behaviour, Sensory processing

## Abstract

Temporal alignment is often viewed as the most essential cue the brain can use to integrate information from across sensory modalities. However, the importance of conscious perception of synchrony to multisensory integration is a controversial topic. Conversely, the influence of cross-modal incongruence of higher level stimulus features such as phonetics on temporal processing is poorly understood. To explore the nuances of this relationship between temporal processing and multisensory integration, we presented 101 participants (ranging from 19 to 73 years of age) with stimuli designed to elicit the McGurk/MacDonald illusion (either matched or mismatched pairs of phonemes and visemes) with varying degrees of stimulus onset asynchrony between the visual and auditory streams. We asked them to indicate which syllable they perceived and whether the video and audio were synchronized on each trial. We found that participants often experienced the illusion despite not perceiving the stimuli as synchronous, and the same phonetic incongruence that produced the illusion also led to significant interference in simultaneity judgments. These findings challenge the longstanding assumption that perception of synchrony is a prerequisite to multisensory integration, support a more flexible view of multisensory integration, and suggest a complex, reciprocal relationship between temporal and multisensory processing.

## Introduction

The brain’s ability to integrate information from different sensory modalities is essential to making sense of a complex perceptual environment. Multisensory integration has been shown to enhance perception^1,2^, reduce ambiguity^3–5^, and even produce powerful illusions^6–8^. In all of these cases, different types of congruence across sensory modalities increase the likelihood that integration will occur. The most frequently studied are consistencies in space and time, which are perhaps the two most obvious physical features the brain could use to bind together relevant sensory input coming from a common source. Accordingly, spatiotemporal alignment has been found to enhance the likelihood of multisensory integration in numerous contexts^9^. While both factors play an important role, research has also shown that temporal synchrony across modalities alone can be sufficient to enhance perception^10–12^, and even elicit striking illusions like the McGurk/MacDonald effect^8^ and double-flash illusion^6,7^. For these reasons, temporal synchrony has often been viewed as a sort of glue that binds multisensory information together, and as the type of cross-modal consistency most essential to integration^10^.

Of the illusions mentioned, one of the most extensively studied is the McGurk/MacDonald effect, wherein the presentation of a mismatched phoneme (the auditory component of a syllable) and viseme (its visual counterpart) leads to the perception of a syllable distinct from either the visual or auditory input, a phenomenon dubbed a “fusion,” which is considered a textbook case of multisensory integration. For example, in the classic study for which the effect is named, the presentation of a/ba/ phoneme along with a/ga/ viseme produced an illusion in which participants reported that they heard/da/ with striking frequency^8^. The illusion has measurable temporal constraints and occurs more frequently when the auditory and visual stimuli are presented closer to synchrony or with a very slight visual lead^13^. This makes perfect sense, as the transduction of auditory signals is much quicker than that of visual ones^14^, which means that audiovisual integration is often optimized with a slight visual lead^10,15^.

While timing is clearly essential to the McGurk/MacDonald illusion, controversy exists as to whether conscious perception of synchrony is necessary to its elicitation. Perception of temporal synchrony is often thought to be key to the assumption of unity, or the impression that two stimuli come from a common source^16^. The Unity Assumption Hypothesis holds that this common source judgment has a considerable influence on multisensory interactions^17,18^. It states that the brain will be more likely to treat information from different senses with greater similarity in amodal properties (particularly temporal coincidence) as emanating from a common source, allowing integration^16^. In line with this view, one would expect perception of synchrony to be essential to the McGurk/MacDonald illusion, as two stimuli that are noticeably occurring at different times clearly cannot belong to the same event.

However, several studies have challenged this long-standing assumption. Soto-Faraco and Alsius^19^ found that when participants were asked to perform both temporal order judgments (TOJ) and respond to McGurk/MacDonald stimuli, on trials in which they noticed that the sound led the video by 240 or 160 ms, they still experienced the illusion 59% of the time. However, temporal order judgments force a choice between one stimulus or the other leading, and are subject to bias^20^. Therefore, they are not well equipped to study exactly when something is or is not perceived as simultaneous. To account for this, in a second study^21^, participants were asked to perform the McGurk/MacDonald task in addition to a simultaneity judgment (SJ) task. With SJs, participants are even more acutely sensitive to subtle temporal asynchronies, providing more direct insight into when stimuli are perceived as simultaneous. The findings from the second study were compelling: the window in which the McGurk/MacDonald effect occurred was significantly wider than that in which the stimuli were recognized as simultaneous (which we will refer to as the window of perceived synchrony, or WPS), and the illusion still occurred frequently on trials in which participants reported that the stimuli were not synchronized. The authors took this as evidence for a more flexible view of multisensory integration, wherein different attributes of stimuli can be recognized and combined at different times, and multisensory integration can occur despite recognition of temporal misalignment. These conclusions challenge more traditional interpretations of the Unity Assumption Hypothesis, including the notion that perception of temporal synchrony is a prerequisite to multisensory integration.

However, the WPS calculated with incongruent stimuli may be narrower than with congruent stimuli, which complicates interpretation of this finding and has led to differing conclusions. Vatakis and Spence^22^ interpreted such a finding as an improvement in temporal processing resulting from violation of the unity assumption. Relatedly, van Wassenhove, Grant, and Poeppel^23^ found that, in separate SJ and McGurk/MacDonald tasks, incongruent McGurk/MacDonald stimuli were perceived as synchronous significantly less frequently across a range of SOAs. The findings of both of these studies could be taken as evidence that participants are more accurate at performing TOJ and SJ tasks with mismatched audio-visual stimuli because they are not being bound, and the stimuli are less likely to be bound due to the incongruence working against the assumption of unity. However, a conspicuous issue with this interpretation is the fact that SJ rates were far lower for incongruent trials even when they were actually physically simultaneous in van Wassenhove, Grant, and Poeppel^23^. Participants went from recognizing the physical simultaneity 98% of the time to a mere 74% of the time. This cannot be construed as an improvement of accuracy afforded by incongruence and is actually a striking interference with processing of temporal relationships. The interference in temporal processing posed by phonetic incongruence challenges the predominant view of temporal processing as an immutable foundation that higher level multisensory interactions (like phonetic congruence/incongruence of the audiovisual streams) cannot influence significantly.

Taken together, these findings provide complex and contradictory portrayals of the nature of the relationship between temporal dynamics and multisensory perception, particularly with regard to the McGurk/MacDonald effect. Each study makes compelling arguments; however, ultimately, none of them are equipped to fully disentangle the alternatives by virtue of their design and analytic approaches. Separate temporal and McGurk/MacDonald tasks (as in van Wassenhove, Grant, and Poeppel^23^) do not allow one to directly assess if the illusion is in fact occurring when stimuli are not perceived as synchronous. This leaves open the possibility that the effect of incongruence on synchrony judgments was driven by trials in which the McGurk/MacDonald illusion was not elicited, which would support the Unity Assumption Hypothesis. Soto-Faraco and Alsius^21^ does speak to the former issue, but does not analyse the influence of congruency on synchrony distributions, again leaving questions as to what exactly is driving the effect of incongruence on temporal judgments, and what it means for the debate surrounding the Unity Assumption Hypothesis. If the difference in synchrony response rates between congruent and incongruent stimuli is not in fact resulting from trials in which the illusion fails, this would serve as evidence against traditional interpretations of the Unity Assumption Hypothesis, and would support Soto-Faraco and Alsius’s more flexible view of multisensory integration. To understand how these findings speak to the Unity Assumption Hypothesis, both analytical angles must be taken into account. We must both consider whether perception of synchrony is necessary to the illusion, and whether (lack of) perception of the illusion is what is driving the differences in synchrony judgments. To answer the question of whether multisensory processes like the McGurk/MacDonald illusion are indeed cognitively irreducible, unitary phenomena, to reconcile these conflicting perspectives on the importance of assumption of unity to integration, and to better understand the effect of (in)congruence across sensory modalities on temporal judgments, we have conducted a new study, which fuses the strengths of prior research and allows novel analytical approaches to these heretofore intractable problems.

## Methods

### Participants

101 participants were recruited via Prolific Academic (53 males, 48 females; mean age was 30.64 years, ranging from 19 to 73 years). The participants gave informed consent prior to the experiment, received £15 for their participation (in this experiment, as well as several others included in a broader test battery), and were naïve as to the purpose of the experiment. The experiment was approved by the ethical committee from the Vrije Universiteit Amsterdam (VCWE-2020-041R1) in accordance with all guidelines and regulations as specified in the Netherlands Code of Conduct for Research Integrity.

### Apparatus and stimuli

The experiment was programmed and run using Neurotask (www.neurotask.com). The participants performed the experiment online using their own computer and keyboard to perform the task. A movie of an actress saying the syllable /ga/ was recorded^24^. The movie was edited such that the audio information was either congruent (hearing /ga/ and viewing /ga/) or incongruent (hearing /ba/ and viewing /ga/). Moreover, the stimulus onset asynchrony (SOA) between the lip-movements and the voice was manipulated: − 500, − 260, 0, 260, or 500 ms. Here, negative values indicate that the voice was leading the lip-movements, and vice versa. All movies were shown for 2000 ms.

### Procedure and design

**Figure 1:**
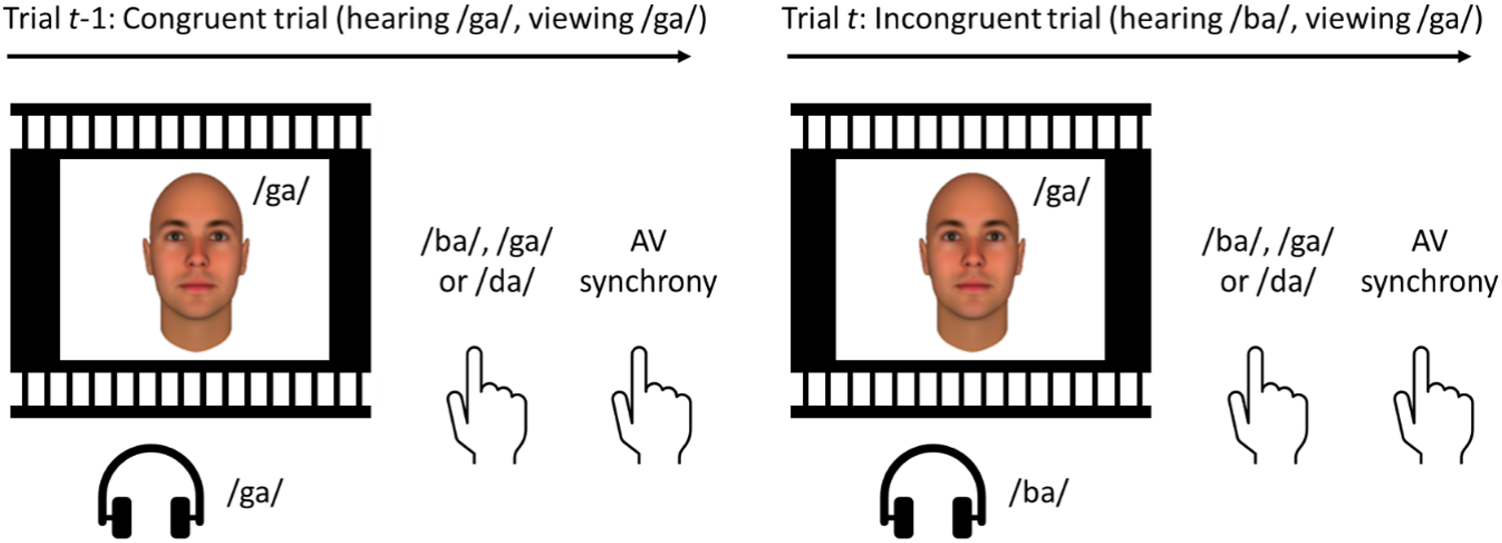
Two illustrative example trials used in the study. The participants viewed a movie of an actress mouthing the syllable /ga/. On half the trials, audio corresponded to the video (i.e., congruent trials), whereas on the remaining trials, the audio of the syllable /ba/ was played (i.e., incongruent trials). The onset between the voice and the lip movement was manipulated. Participants were instructed to report whether they heard /ba/, /ga/, or /da/. Subsequently, they were instructed to judge whether the voice was synchronized with the lip movements. Note: a computer generated face overlays the actress for privacy.

A trial started with the presentation of a black fixation cross at the centre of a white background for 1000 ms, followed by the movie for 2000 ms (see Fig. 1). On half of the trials, the voice was congruent with the lip-movements, whereas on the remaining trials, they were incongruent. After the movie, participants were instructed to make two self-paced responses. Firstly, they reported whether they heard /ba/, /ga/, or /da/ by pressing the b-, g-, or d-key, respectively (note that participants did not respond to whether the viseme/phoneme matched, simply to which syllable they perceived). Secondly, they reported whether the lip movement was synchronized with the voice or not by pressing the 1- or 0-key, respectively. The next trial was initiated after participants made the synchrony judgment. There were 100 trials in total (2 congruencies × 5 SOAs × 10 repetitions) and the order was randomly determined. Participants received written instructions prior to the experiment and practiced all the different conditions once (i.e., 10 trials) to get familiar with the stimuli and both tasks. The experiment lasted ~ 7 min and was part of a battery of online experiments (not reported here).

## Results

The data from eleven participants were excluded from further analyses, since their performance matched one or multiple exclusion criteria, as pre-registered in As Predicted (#102341). More specifically, one participant was excluded for opening other applications on their browser (like facebook, email, etcetera) on more than 10% of the trials during the experiment. Two participants were excluded because they did not complete the experiment. Eight participants were excluded because their perceived synchrony distributions on congruent trials were non-Gaussian (i.e., the mean proportion perceived synchrony was greater than 0.5 for the − 500 ms SOA and the amplitude difference across SOAs was smaller than 0.4). Five participants were excluded since they responded with /ba/ or /da/ on more than 25% of the time on congruent trials.

### Synchrony judgements

**Figure 2:**
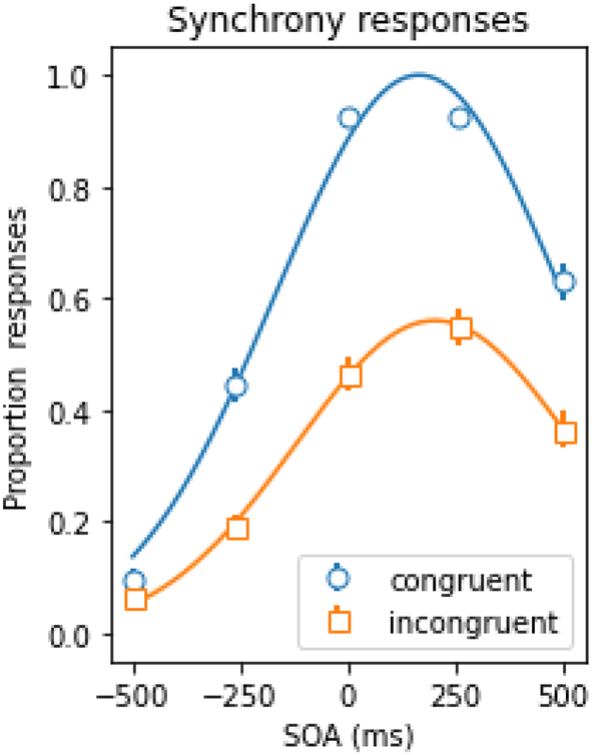
Mean proportion of synchrony responses as a function of the stimulus onset asynchrony (SOA) for congruent and incongruent trials. Here, negative SOAs indicate that the voice was leading the lip movements, and vice versa. The continuous lines represent the best Gaussian fit (r^2^ ≥ 0.99). The error-bars reflect the standard error of the mean.

Figure 2 illustrates the mean proportion of synchrony responses as a function of the SOA for congruent (blue circles) and incongruent trials (orange squares).

We conducted a repeated measures ANOVA on the mean proportion of synchrony responses with SOA and congruency as within subject variables using Just Another Statistical Program (JASP)^25^. Here, and elsewhere in the manuscript, alpha was set to 0.05, and *p* values were Hyunh-Feldt corrected to avoid sphericity violations. Additionally, for all parametric tests, here and elsewhere throughout the manuscript, normality of the distributions was assessed. In cases where the assumption of normality was violated and significant findings were detected, follow up Friedman tests were employed as non-parametric alternative. The ANOVA yielded a significant main effect for SOA, *F*(4, 352) = 255.758, *p* < 0.001, $\eta_{p}^{2}$  = 0.744, as the proportion of synchrony responses depended on the SOA (i.e. a typical Gaussian distribution around 0 ms, see e.g.^26–28^). Interestingly, the congruency main effect was significant, *F*(1, 88) = 213.266, *p* < 0.001, $\eta_{p}^{2}$  = 0.708, as the overall proportion synchrony responses dropped from 0.60 for congruent trials to 0.33 for incongruent trials. The SOA × congruency interaction was also significant, *F*(4, 352) = 42.534, *p* < 0.001, $\eta_{p}^{2}$  = 0.326, as the magnitude of the congruency effect depended on the SOA. Larger congruency effects were observed when the voice and lip movement were approximately synchronized and a smaller effect was observed when the SOA was − 500 ms, since participants performed almost at ceiling (i.e., they never perceived the stimuli as being synchronous for such a long SOA). However, as the assumption of normality was violated for synchrony distributions, we followed up with a series of Friedman tests. These confirmed a significant effect of congruence collapsed across SOAs (*x*^2^(1) = 74.711, *p* < 0.001). We also detected a significant effect of SOA on synchrony judgments for both congruent (*x*^2^(4) = 281.794, *p* < 0.001) and incongruent trials (*x*^2^(4) = 188.681, *p* < 0.001), as well as a significant effect of SOA on the difference between congruent and incongruent synchrony judgment response rates (*x*^2^(4) = 121.869, *p* < 0.001), confirming the SOA × congruency interaction.

### McGurk/MacDonald effect

**Figure 3:**
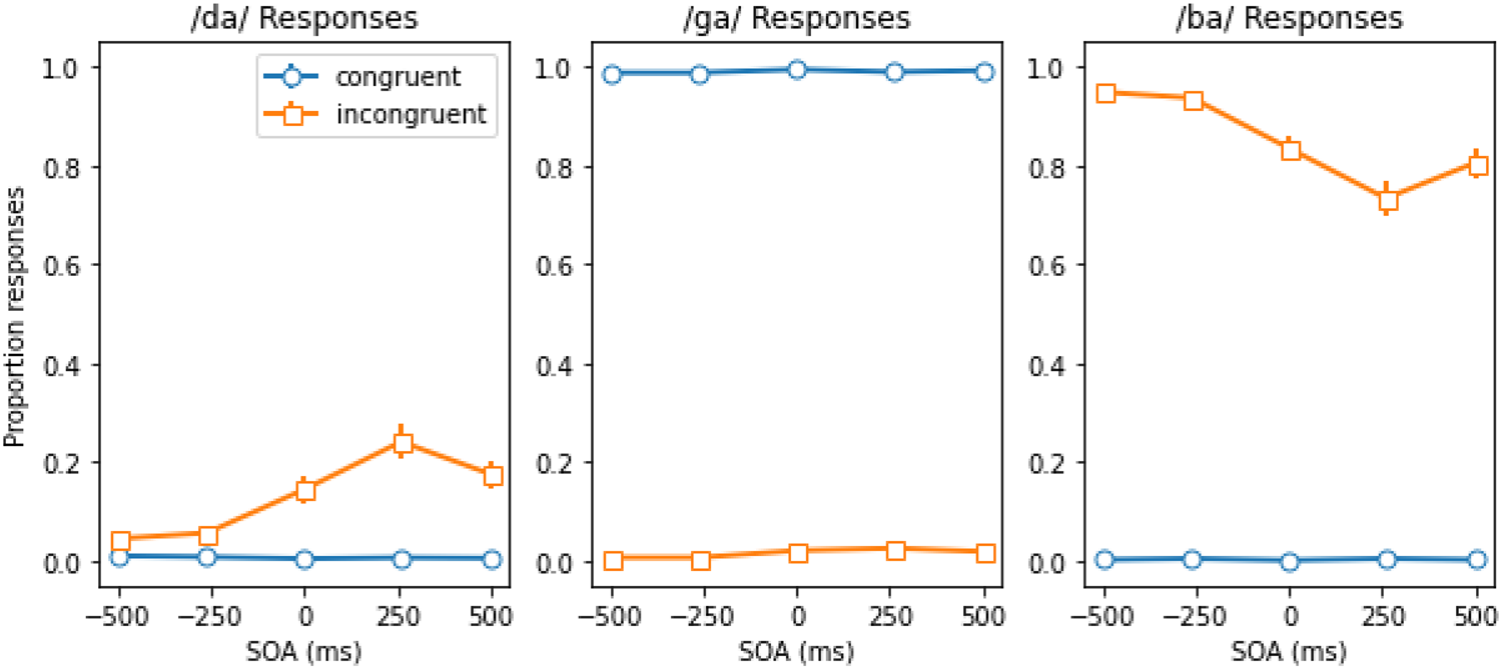
From left to right, the mean proportion of /da/, /ga/, and /ba/ responses as a function of the stimulus onset asynchrony (SOA) for congruent and incongruent trials. Here, negative SOAs indicate that the voice was leading the lip movements, and vice versa. The error-bars reflect the standard error of the mean.

The mean proportions of /da/, /ga/, and /ba/ responses as a function of the stimulus onset asynchrony (SOA) for congruent and incongruent trials are shown in Fig. 3 (from left to right).

We conducted an ANOVA on the mean proportion of /da/, /ga/, and /ba/ responses with SOA and congruency as within subject variables. The ANOVA on the mean proportion of /da/ responses (i.e., fusion trials) yielded a significant congruency effect as well as a significant congruency × SOA interaction, *F*(1, 89) = 42.240, *p* < 0.001, $\eta_{p}^{2}$  = 0.326, and *F*(4, 356) = 22.910, *p* < 0.001, $\eta_{p}^{2}$  = 0.205, respectively. The two-way interaction indicates that the congruency effect depended on the SOA between the voice and the lip movements. Although it is clear that the congruency effect (the proportion of /da/ responses for incongruent trials—the proportion of /da/ responses for congruent trials) was larger when the SOA was 0, 260, or 500 ms compared to the -500 and -260 ms conditions, separate two-tailed *t*-tests yielded a significant congruency effect for each SOA condition (all *t*(89) values ≥ 2.758, all *p* values ≤ 0.007). In other words, a significant McGurk/MacDonald effect was observed for each SOA condition, but the magnitude depended on the asynchrony between the lip-movements and the voice. The main effect of SOA was also significant, *F*(4, 356) = 20.817, *p* < 0.001, $\eta_{p}^{2}$  = 0.190, but not examined in further detail. As the assumption of normality was violated for /da/ response rates, we conducted follow up Friedman tests. These confirmed the effect of congruency on /da/ response rates was significant both collapsed across SOAs (*x*^2^(1) = 38.754, *p* < 0.001) and at each specific SOA (all *x*^2^(1) values ≥ 7.538, all *p* values ≤ 0.006). They also showed that that SOA had a significant effect in incongruent trials (*x*^2^(4) = 65.907, *p* < 0.001). SOA did not, however, have a significant effect on congruent trials (*x*^2^(4) = 2.250, *p* = 0.690), suggesting an interaction between congruency and SOA that was confirmed by testing the significance of the effect of SOA on the difference in /da/ response rates between incongruent and congruent trials (*x*^2^(4) = 72.200, *p* < 0.001).

The ANOVA on the mean proportion of /ga/ responses yielded a significant congruency effect, *F*(1, 89) = 49,072, *p* < 0.001, $\eta_{p}^{2}$  = 0.998, indicating that participants perceived /ga/ when both the lip movements and the voice were making /ga/ on the vast majority of trials (99%), but not when the audio of the syllable /ba/ was played (1%). The main effect of SOA was significant, *F*(4, 356) = 3.301, *p* = 0.019, $\eta_{p}^{2}$ = 0.036, but not further examined. The two-way interaction failed to reach significance, *F*(4, 356) = 1.420, *p* = 0.236, $\eta_{p}^{2}$  = 0.016. Follow up Friedman tests revealed a significant effect of SOA on incongruent trials (*x*^2^(4) = 10.642, *p* = 0.031), but not on congruent ones (*x*^2^(4) = 3.535, *p* = 0.473), where participants almost always perceived /ga/. Congruency also had a significant effect on /ga/ responses collapsed across SOAs (*x*^2^(1) = 90.000, *p* < 0.001).

The ANOVA on the mean proportion /ba/ responses yielded a significant main effect of congruency, *F*(1, 89) = 1766 *p* < 0.001, $\eta_{p}^{2}$  = 0.952, as the participants perceived /ba/ on the majority of trials when the audio of the syllable /ba/ was played (incongruent trials), but not when the /ga/ sound was played (congruent trials). This effect depended on the SOA, as the two-way interaction was highly significant, *F*(4, 356) = 25.854, *p* < 0.001, $\eta_{p}^{2}$  = 0.225. In other words, on incongruent trials, participants almost always perceived /ba/ if they did not perceive the McGurk/MacDonald effect (see the opposite pattern in Fig. 3, comparing the left to the right panel). The main effect of SOA was again significant, *F*(4, 356) = 25.238, *p* < 0.001, $\eta_{p}^{2}$  = 0.221. Here, follow up Friedman tests confirmed the significance of the effect of congruency on /ba/ responses collapsed across SOAs (*x*^2^(1) = 90.000, *p* < 0.001). They also showed that SOA had a significant effect on /ba/ responses for incongruent trials (*x*^2^(4) = 66.203, *p* < 0.001). Finally, they revealed a significant effect of SOA on the difference in /ba/ response rates between incongruent and congruent trials (*x*^2^(4) = 62.413, *p* < 0.001), confirming the interaction detected in the ANOVA.

### Rapid temporal recalibration

To measure rapid temporal recalibration, we binned the trials based on the modality order on the previous trial. Subsequently, we fitted a Gaussian function^26–28^ to the synchrony distribution for each condition (audition was leading on the previous trial, or vision was leading on the previous trial) to estimate the point of subjective simultaneity (PSS), which reflects the asynchrony where the stimuli are most likely to be perceived as simultaneous. We found evidence for rapid temporal recalibration^26–28^ by showing that the PSS was contingent upon the modality order of the preceding trial. More precisely, the PSS was significantly smaller when audition led in it (156 ms) than when vision did (192 ms), as was revealed in a two-tailed *t* test (*t*(89) = 3.104, *p* = 0.002). See Supplementary Fig. 1 for more information.

### Synchrony Judgments

**Figure 4:**
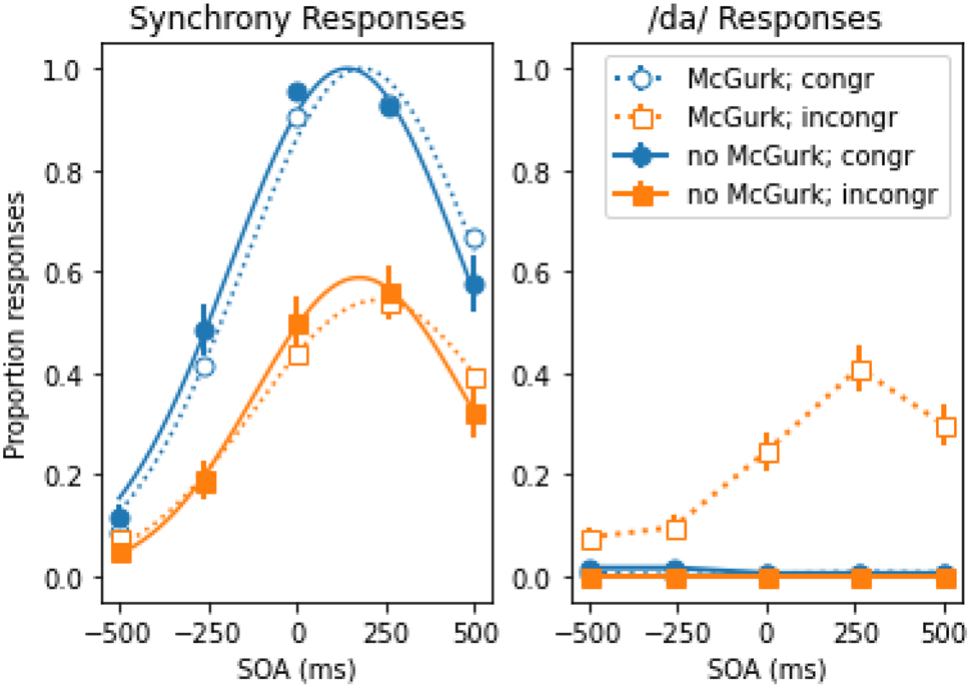
Mean proportion of synchrony responses as a function of the stimulus onset asynchrony (SOA) and congruency for participants who did not perceive the McGurk/MacDonald effect (solid line; n = 37) and participants who perceived the McGurk/MacDonald effect (dotted line; n = 53). Here, negative SOAs indicate that the voice was leading the lip movements, and vice versa. The continuous lines represent the best Gaussian fit (r^2^ ≥ 0.98). The error-bars reflect the standard error of the mean.

Beyond what was pre-registered, we conducted several exploratory analyses to better understand the nuances of the effects we detected. The previous synchrony analyses revealed a precipitous drop in the mean proportion of synchrony responses for incongruent trials compared to congruent trials. The obvious question, then, is why the proportion of synchrony responses is so low for incongruent trials if participants integrate the auditory and visual event into a single unified fusion percept. A plausible explanation for this drop is that participants did not perceive the McGurk/MacDonald effect on the majority of trials (see also Fig. 3, leftmost panel). If the congruency effect (the difference in the rate of synchrony responses between the congruent and incongruent conditions) is indeed contingent upon observing the McGurk/MacDonald effect, then we would expect a large group difference if we divided the group by whether participants perceived the McGurk/MacDonald effect or not. Therefore, we conducted a post-hoc analysis to examine whether the congruency effect observed in Fig. 1 depended on whether participants perceived the McGurk/MacDonald effect or not by comparing the mean proportion of synchrony responses for participants who did and did not perceive the illusion. Figure 4 (left panel) illustrates the mean proportion of synchrony responses as a function of SOA and congruency for participants who did not perceive the McGurk/MacDonald effect at all (solid lines) and for participants who did perceive it (dotted lines). Figure 4 (right panel) illustrates how often participants perceived the McGurk/MacDonald effect (i.e., proportion /da/ responses) as a function SOA and congruency for both groups.

We conducted an exploratory repeated measures ANOVA on the mean proportion of synchrony responses with SOA and congruency as within subject variables and group (perceived vs did not perceive fusions) as a between subject variable. Note that 53 participants perceived fusions on at least some trials (i.e., mean proportion /da/ responses > 0), and 37 participants never perceived a fusion (i.e., mean proportion /da/ responses = 0). The ANOVA yielded no significant group effect, *F*(1, 88) = 0.016, *p* = 0.901, $\eta_{p}^{2}$  < 0.001, and there was neither an interaction between group and congruency, *F*(1, 88) = 0.140, *p* = 0.709, $\eta_{p}^{2}$  = 0.002, nor between group and the congruency × SOA interaction, *F*(4, 352) = 0.794, *p* = 0.511, $\eta_{p}^{2}$  = 0.009, indicating that the congruency effect was not dependent on whether participants perceived the McGurk/MacDonald effect.

**Figure 5:**
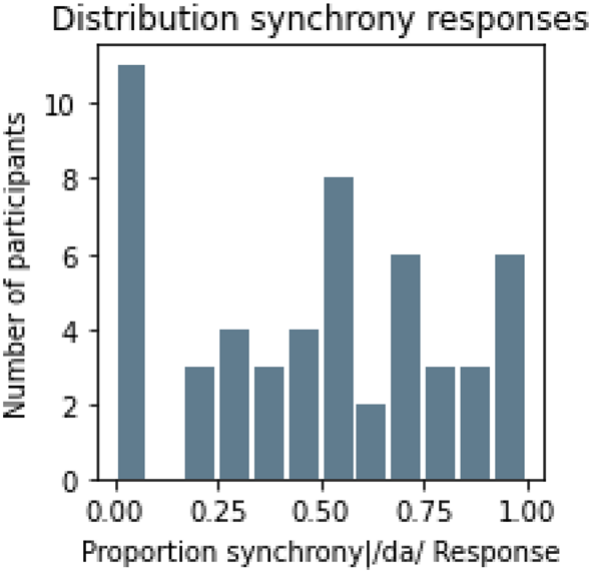
Number of participants as a function of the perceived synchrony for those trials in which the McGurk/MacDonald effect was perceived (i.e., /da/ responses only).

In an exploratory analysis, to ascertain whether the McGurk/MacDonald effect could in fact occur despite participants perceiving the stimuli as asynchronous, we analysed the number of participants as a function of the perceived synchrony for those trials in which the McGurk/MacDonald effect was perceived (i.e., /da/ responses only), as seen in Fig. 5. This figure illustrates that this was the case; in fact, the mean proportion of synchrony judgments was less than 50% over all participants (i.e., 0.466) on trials in which a fusion occurred.

In a final exploratory analysis, we sought to investigate the relationship between perception of the illusion and perception of synchrony more directly. To circumvent the confounding influence of SOA on both judgments, we held it constant, looking specifically at incongruent (McGurk/MacDonald) trials from the SOA most likely to produce both the illusion and a positive synchrony judgment (260 ms). It was common here for participants to either always experience synchrony or never experience the illusion, rendering comparisons between trials in which the illusion/synchrony were/were not experienced impossible for some. This resulted in the exclusion of 11 participants (for a new total of 79) for the fusion rate analysis and the exclusion of 49 participants (for a total of 41) for the synchrony judgment rate analysis. We then conducted Friedman tests to determine whether the relationship between synchrony judgments and fusion rates was significant in either direction. When comparing the rate of the McGurk effect on trials in which synchrony was experienced (27% of the time) to those in which it was not (22% of the time) for the 79 participants who experienced both asynchrony and synchrony at least once at the 260 ms SOA, we found that the difference was not significant (*x*^2^(1) = 0.030, *p* = 0.862)*.* Conversely, when comparing the rate of synchrony judgments on trials in which the McGurk effect was experienced (58%) to those in which it was not (50%) for the 41 participants who both did and did not experience the illusion at least once at the 260 ms SOA, we found that this difference was also non-significant (*x*^2^(1) = 0.030, *p* = 0.862)*.*

Note that there was one pre-registered analysis that we did not conduct. This analysis was intended to compare the width of the WPS across trial types to the rate of the illusion. However, due to the large difference in synchrony judgment rates between congruent and incongruent trials, this analysis was not sensible.

## Discussion

Our results have spoken directly to a lasting controversy regarding the relationship between perception of synchrony and multisensory integration as seen in the McGurk/MacDonald illusion. First, we have replicated prior findings demonstrating that the effect is highly dependent on timing, and is optimally produced when the auditory and visual streams are presented close to simultaneity, with a slight visual lead^13^. This pattern was also reflected in synchrony judgments for both congruent and incongruent trials. While there is clearly a similarity in these patterns, we have also shown compelling evidence for a distinction between perception of synchrony and perception of the illusion.

To begin, like van Wassenhove, Grant, and Poeppel^23^, we found that participants were much less likely to perceive synchrony on the incongruent trials than they were on the congruent ones. This is a striking finding due to the dramatic effect that a higher level difference in phonetic properties appears to have had on basic temporal processing, which is often seen as a foundation for multisensory integration^16,29^. It is curious, given the longstanding assumption that perception of synchrony is very important (if not necessary) for the McGurk/MacDonald effect to occur, that the very stimuli that elicit it have been shown to interfere so drastically with recognizing simultaneous stimuli.

There are several possible interpretations of this congruency effect. Van Wassenhove, Grant, and Poeppel^23^ attributed it to the relationship between the acoustic dynamic envelope and facial kinematics, which is closer in the case of the congruent stimuli. They concluded that this was reason to anticipate that at the same SOA, an incongruent pairing would be less likely to be judged simultaneous than a congruent one. However, this explanation focuses more on how the features of the stimuli differ than the exact manner in which these discrepancies translate into differences in perception and behaviour. As such, the way in which they might influence temporal processing is unclear. That said, the acoustic dynamic envelope and facial kinematics do share a very intimate relationship built over years of experience with matching phonemes and visemes that is violated on incongruent trials. This visuo-phonological association is reflected in an extensively distributed cortical network that is a recent topic of research^30^, and its possible involvement in temporal processing has not been assessed. Accordingly, designs in which the disparity between the acoustic dynamic envelope and associated facial kinematics is manipulated more directly and precisely along with SJs are necessary to further examine the feasibility of this explanation.

An alternative explanation might follow the lines of Vatakis and Spence^22^, asserting that incongruence was leading to an improvement in performance in temporal judgments. This would provide support for the Unity Assumption Hypothesis due to the fact that asynchrony was detected more frequently across the range of SOAs presented for incongruent trials (which would be less likely to be attributed to a common source). However, this interpretation is at odds with the fact that the effect of incongruence is still very strong at both actual physical synchrony and with a slight visual lead, where stimuli are normally perceived as synchronous. Accordingly, our findings cannot be interpreted as an improvement of performance, but rather as interference. In this way, the cause of the incongruence effect seen in our study and that of van Wassenhove and Poeppel^23^ does not appear to be satisfactorily explained by the Unity Assumption Hypothesis. Rather, it is more in line with a flexible perspective on multisensory processing, wherein temporal integration and multisensory integration are separate but related processes to which individuals have some degree of independent conscious access, as was concluded in Soto-Faraco and Alsius^19^.

There are also alternative interpretations as to the level at which the influence of incongruence on temporal processing occurs. As multisensory interactions, especially those involving the influence of visual input on auditory processing, have been shown to occur very early in sensory processing^31^, it is possible the influence occurs at a very early sensory level. Additionally, the superior temporal sulcus is a region key to audiovisual interactions, particularly with regard to speech stimuli. It has been associated with perception of the McGurk/MacDonald illusion^32^ and has also been shown to react differently to synchronous and asynchronous audiovisual speech stimuli^33^. It is therefore possible that the influence of incongruence on perception of synchrony could occur at a higher multisensory hub. Alternatively, it is possible that a higher level cognitive influence explains the effect. Incongruent McGurk/MacDonald stimuli are often described as feeling “off” or “awkward” even when fused, so it is possible that participants might be biased against rating them as synchronous. It is very difficult to rule out this sort of interpretation with a task that is inherently subjective. However, two factors weigh against this interpretation, at least as a full explanation. The manner in which the magnitude of the effect scales with SOA suggests a relationship with temporal processing that is more intimate than a simple response bias. Additionally, both those who perceive the illusion and those who do not perceive it experience the incongruence effect to the same degree. Conscious perception of the illusion would be expected to have some influence on feelings of awkwardness, as it would lead to a resolution of the conflict between what is seen and heard. However, it seems the physical features of the stimuli themselves, and not conscious perception of them, drive the effect, which is at odds with a top-down bias interpretation. In any case, it is clear that there is a significant influence of incongruence on perception of synchrony. Future research should focus on disentangling these alternatives, identifying the mechanism of the interaction, and pinpointing the level at which this effect on temporal processing is occurring.

We also replicated Soto-Faraco and Alsius’s^21^ findings that participants were able to perceive the illusion even in instances where synchrony was not perceived. In fact, across all participants, the average rate at which individuals experienced synchrony when fusions occurred was under 50%, and the likelihood that the illusion would occur was not significantly influenced by perception of synchrony, and vice versa. This provides strong evidence against the necessity of perception of synchrony to multisensory integration, and further support for Soto-Faraco and Alsius’s more flexible view of multisensory processing. This is not to say that temporal alignment is not essential to the illusion; clearly, SOA had a very significant impact on fusion rates, and the illusion ceases to occur when stimuli are sufficiently asynchronous. However, when holding SOA constant, perception of synchrony was not shown to influence the rate of the illusion occurring (and vice versa) for those participants who were susceptible to the effect. This suggests that conscious perception of synchrony is not a strict prerequisite to it and, conversely, that the very incongruence that produces the illusion (but not conscious perception of it) may also interfere with temporal processing.

In this way, by considering both the effect of perceived simultaneity on fusion rates, and of perception of fusion on synchrony distributions, we are finally able to speak to our main questions. Due to our findings that the illusion occurred even without perception of synchrony, and incongruence drove synchrony response rates down irrespective of whether or not the illusion was perceived, Soto-Faraco and Alsius’s^19^ conclusions are further supported. If the incongruence effect were simply explained by trials in which the illusion was not perceived, this may serve as evidence against their conclusions, and their implications with regard to the Unity Assumption Hypothesis; however, this clearly was not the case. Even when the incongruent stimuli were integrated and perceived as a fused percept, there was still a considerably lesser likelihood that they would be judged as simultaneous than there was for congruent stimuli.

While the virtues of our design and comparatively large sample size allow many firm conclusions from our findings, there are of course limitations. First, the fact that this experiment was part of a larger battery of tasks meant that we were limited in the amount of time we could allot to it, placing a constraint on the number of conditions and trials we could present. This meant that function fitting approaches more sensitive to the asymmetry of SJ distributions reported by Yarrow, Solomon, Arnold, and Roseboom (in press)^38^ would have overfit our data (where we have five SOAs, only one more than the number of parameters in the simplest model described). These approaches use more parameters to better capture the malleability of the decision criteria involved in SJs, which they showed to be subject to strategic biases. As such, they may better reflect the true nature of the decision making processes involved, a claim supported by their better predictive performance in their design. While flexible individual biases surely exist in SJ performance, the excellent fit of our Gaussian distributions (minimum r^2^ > 0.98) suggests that with a sufficiently large sample and consistent task demands, they appear to average out, and the synchrony distribution assumes a remarkably symmetrical shape. That being said, future research interested in capturing the nuances of SJ tasks in greater detail and studying the flexibility of the decision criteria involved should involve a wider range of SOAs and more complex models than our design permitted. Conversely, studies not interested in studying these factors would do well to recognize the risk that overfitting data may result in their model incorporating them, particularly when a small sample size allows individual biases to have a more significant influence on overall trends.

An additional benefit of expanding the range of SOAs and trials is that it would allow one to better evaluate whether rapid temporal recalibration can influence the rate at which the McGurk/MacDonald effect occurs and, conversely, whether phonetic incongruence attenuates the influence of rapid temporal recalibration due to its apparent interference in temporal processing. Van der Burg, Alais & Cass^28^ demonstrated that recalibration depends on actual asynchrony, rather than subjective perception, which suggests that recalibration may be unaffected by the incongruence effect observed here, if it exists at a higher cognitive level. However, if the incongruence affects sensation at an early level and more directly distorts temporal processing, one would expect it to similarly interfere with temporal recalibration. In this way, such a design could also speak to the earlier questions regarding the level at which the incongruence effect occurs.

One might also criticize the low rate of McGurk/MacDonald fusions that we produced across our SOAs, and attribute our findings with regard to temporal processing to the fact the illusion was infrequently elicited. However, McGurk/MacDonald fusion rates have been found to be highly variable across designs, and the rate at which we produced the illusion falls comfortably within the range previously observed in the literature^34,35^. Moreover, the rate at which the illusion was produced in our design was not only statistically significant, it varied significantly as a factor of SOA, peaking with a slight visual lead, as in Munhall et al.^13^. This resonates with previous findings, supporting the notion that the illusion was being produced effectively.

An intriguing explanation as to why we encountered fewer fusions than in some previous studies may lie in the linguistic context of the stimuli and our participants. As our cohort was exclusively fluent Dutch speakers, the fact that the Dutch language involves a different /ga/ sound may have played a role. Indeed, studies have shown McGurk/MacDonald susceptibility differs across speakers of different languages, and that Dutch participants may be less subject to the McGurk/MacDonald effect than some other groups^36,37^. This possible explanation for the lower fusion rates prompts another question for future research that might allow us to further examine the relationship between SJ and multisensory integration by comparing across languages. If the more flexible view of Soto-Faraco and Alsius^19^ is accurate, and the window in which the illusion occurs and that in which the stimuli are perceived as synchronous are distinct, while the former should differ across languages, the latter should not.

To conclude, our experiment elucidated much about the nature of the relationship between temporal processing and multisensory integration and spoke to existing controversies regarding the Unity Assumption Hypothesis. Our findings do not contradict its main tenet, that stimuli that share more amodal properties will be more likely to be attributed to the same source and therefore integrated. However, they do provide evidence against interpretations of it that view perception of temporal synchrony as a prerequisite to integration, and they are in line with a more flexible approach to multisensory integration as advanced by Soto-Faraco and Alsius. The surprising manner in which the features that produce the illusion also appear to interfere with temporal integration, but in which its elicitation still depends on some degree of temporal alignment, speaks to the intimate reciprocal relationship between temporal processing and multisensory integration. While this study has resolved some of the questions that inspired its design, it has also opened several new ones. For example, it has called the exact nature of the possible influence of visuo-phonological contrast on temporal integration into question. It has also prompted inquiry into the level at which the incongruence effect occurs (and its possible influence on temporal recalibration) and the manner in which differences in the McGurk/MacDonald effect across languages might illuminate more about the distinction between temporal and multisensory integration. In this way, our study has contributed to the endeavor to better understand the relationship between temporal processing and multisensory integration, an area which has gained more and more interest as dysfunction has been related to various clinical conditions.

### Supplementary Information

Supplementary Figure 1.

## Data Availability

Our raw data includes sensitive personal information and identifiers not suitable for open access. However, upon request to the corresponding author (with a statement of research intent), access to an anonymized data set with all the information necessary to replicate analyses and the Python scripts we used for our analyses will be provided.
