# Supplementary material for: Perception of temporal synchrony not a prerequisite for multisensory integration
Source: Sci Rep. 2024 Feb 29;14:4982. doi: 10.1038/s41598-024-55572-x (PMC10904801; doi:10.1038/s41598-024-55572-x)
Supplement: Supplementary file 1 — Supplementary Figure 1. [file 41598_2024_55572_MOESM1_ESM.docx]

**Perception of Temporal Synchrony Not a Prerequisite for Multisensory Integration (Supplementary Materials)**

Robert M. Jertberg^1^, Sander Begeer^1^, Hilde M. Geurts^2^, Bhismadev Chakrabarti^3,4,5^, and Erik van der Burg^2^

^1^Department of Clinical and Developmental Psychology, The Netherlands and Amsterdam Public Health Research Institute, Vrije Universiteit Amsterdam, Amsterdam, The Netherlands

^2^Brain and Cognition, Department of Psychology, Dutch Autism and ADHD Research Center (d’Arc), Universiteit van Amsterdam, Amsterdam, The Netherlands

^3^Centre for Autism, School of Psychology and Clinical Language Sciences, University of Reading, Reading, UK

^4^India Autism Center, Kolkata, India

^5^Department of Psychology, Ashoka University, Sonipat, India


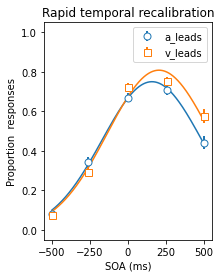


Supplementary Figure 1. Rapid temporal recalibration effect. The mean proportion of responses is shown as a function of stimulus onset asynchrony (SOA) for each modality order condition on the previous trial. Here, negative SOAs indicate that audition led vision, and vice versa.
